# Phase contrast MRI with flow compensation view sharing (FCVS)

**DOI:** 10.1186/1532-429X-16-S1-O7

**Published:** 2014-01-16

**Authors:** Da Wang, Jiaxin Shao, Stanislas Rapacchi, Matthew J Middione, Daniel B Ennis, Peng Hu

**Affiliations:** 1 Radiological Sciences, David Geffen School of Medicine, University of California, Los Angeles, Los Angeles, California, USA; 2 Biomedical Physics Interdepartmental Graduate Program, University of California, Los Angeles, Los Angeles, California, USA

## Background

**Figure 1:**
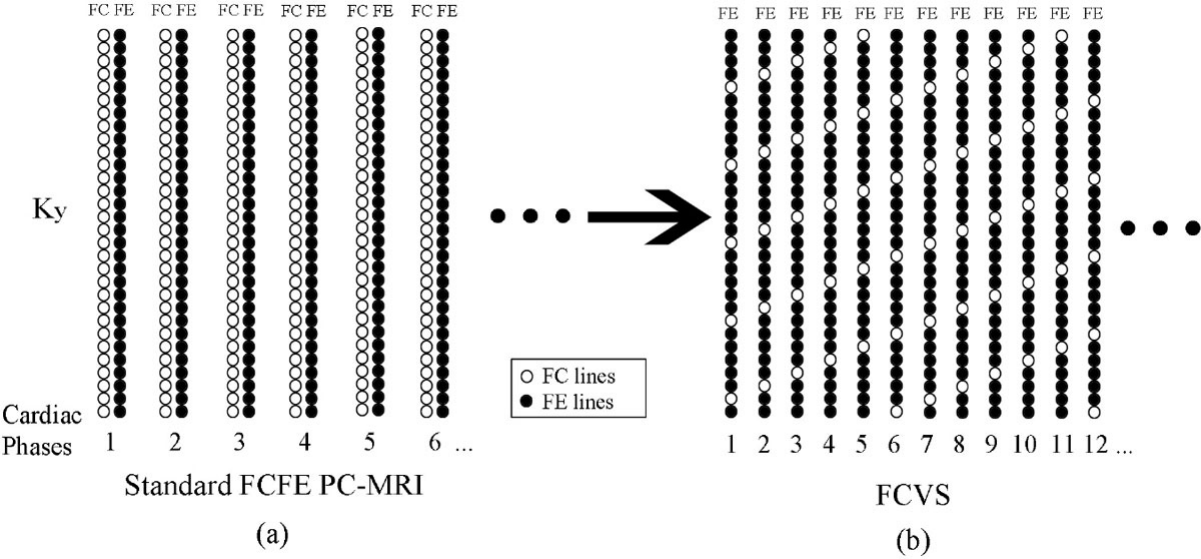
**The data acquisition scheme of (a) the standard FCFE PC-MRI and (b) the proposed FCVS approach**. The FCVS approach approximately doubles the effective temporal resolution by under-sampling the FC data by a factor of six.

Phase-contrast MRI (PC-MRI) is routinely used for quantification of blood flow and velocity in clinical. In a typical PC-MRI exam, flow compensated (FC) and flow encoded (FE) images are alternatively acquired as shown in Figure 1(a). However, in common carotid artery (CCA), FC images are very consistency due to limited physiological motion and background phase changes. In this regard, we propose to accelerate PC-MRI by using sliding window temporal view sharing of the FC data (FCVS) as shown in Figure 1(b). FCVS can improve both the temporal resolution and temporal footprint.

## Methods

Six healthy volunteers were recruited in the prospective study and were scanned by the standard FCFE PC-MRI sequence and FCVS sequence under free breathing. The in vivo study were performed on SIEMENS 1.5 T Avanto scanner with 6-channel head and neck coils. FC k-space lines were acquired after every five FE lines and generated a sliding window under-sampled pattern by a rate RFC = 6. For each corresponding FE frame, a composite FC frame was synthesized by sharing data from adjacent frames. The 5/6 under-sampling of FE data due to FC data acquisition were recovered by TGRAPPA reconstruction. The peak velocity and total volumetric flow measurements of FCVS are compared with standard FCFE with same temporal resolution but double total acquisition time.

## Results

**Figure 2:**
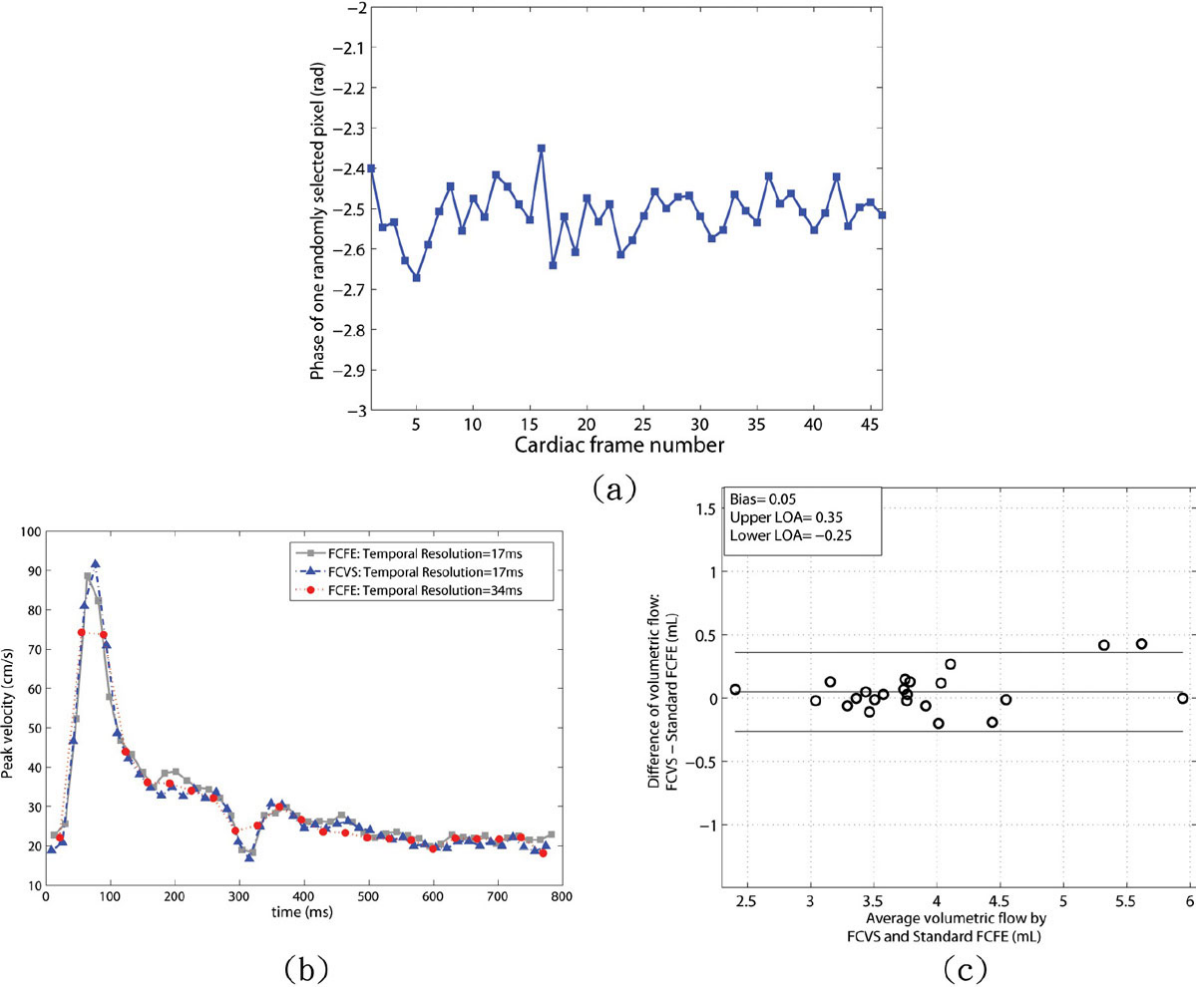
**(a) The FC signal phase as a function of the cardiac frames for a randomly selected pixel within the CCA (mean/± SD: -2.51/± 0.065)**. (b) Peak velocity waveforms from the standard FCFE PC-MRI (gray curve) with 17 ms-temporal-resolution, FCVS (blue curve) with 17 ms-temporal-resolution, standard FCFE PC-MRI (red curve) with 34 ms-temporal-resolution. The FCVS results are highly correlated with the measurements from standard FCFE PC-MRI at the same temporal resolution but FCVS only requires 50% of the acquisition time. The standard FCFE PC-MRI fails to capture the peak velocity at approximately 75 ms or the transient dip at 320 ms when its temporal resolution is halved to match the total acquisition time of FCVS. (c) The Bland-Altman plot of total volumetric flow measurements between standard FCFE PC-MRI and FCVS with two different temporal resolutions (17 ms and 34 ms) in the left and right CCA in six volunteers for a total 24 flow measurement.

The FC signal phase of a randomly selected pixel within a volunteer's CCA (Figure 2a, mean/± SD: -2.51/± 0.065 rad) was stable through cardiac cycle. An example of a healthy volunteer's peak velocity measurements with: 1) FCFE PC-MRI with 2 views-per-segment and 34 ms temporal resolution, 2) FCVS with 2 views-per-segment and 17 ms-temporal-resolution, and 3) FCFE PC-MRI with 1 view-per-segment with 17 ms-temporal-resolution are shown in Figure 2b. The 34 ms-temporal-resolution FCFE scan failed to capture the maximum peak velocity at around 90 ms into the cardiac cycle. The 17 ms FCVS scan provided similar peak velocity values as the 17 ms FCFE scan albeit at half of the total acquisition time. A Bland-Altman plot of total 24 volumetric flow values (left and right CCA in the six volunteers with 17 ms and 34 ms temporal resolutions) measured by FCVS and standard FCFE PC-MRI are shown in Figure 2c. The bias was 0.05 mL and the 95% confidence interval was [-0.25, 0.35] mL. The bias error in volumetric flow quantification was ≤1.3%.

## Conclusions

FCVS can accelerate PC-MRI acquisitions while maintaining flow and velocity measurement accuracy when there is limited temporal variation in the FC data.

## Funding

No funding.

